# Machine learning in cardiovascular magnetic resonance: basic concepts and applications

**DOI:** 10.1186/s12968-019-0575-y

**Published:** 2019-10-07

**Authors:** Tim Leiner, Daniel Rueckert, Avan Suinesiaputra, Bettina Baeßler, Reza Nezafat, Ivana Išgum, Alistair A. Young

**Affiliations:** 1 Department of Radiology | E.01.132, Utrecht University Medical Center, Heidelberglaan 100, 3584CX Utrecht, The Netherlands; 2 Biomedical Image Analysis Group, Department of Computing, Imperial College, London, UK; 3 Department of Anatomy and Medical Imaging, University of Auckland, Auckland, New Zealand; 4 Department of Radiology, University Hospital of Cologne, Cologne, Germany; 5 Institute of Clinical Radiology and Nuclear Medicine, University Medical Centre Mannheim, Medical Faculty Mannheim, Heidelberg University, Mannheim, Germany; 6 Department of Medicine (Cardiovascular Division), Beth Israel Deaconess Medical Centre, Harvard Medical School, Boston, MA USA; 7 Image Sciences Institute, University Medical Center Utrecht, Utrecht, Netherlands; 8 Department of Biomedical Engineering, King’s College London, London, UK

**Keywords:** Cardiovascular magnetic resonance, Machine learning, Deep learning, Radiomics

## Abstract

Machine learning (ML) is making a dramatic impact on cardiovascular magnetic resonance (CMR) in many ways. This review seeks to highlight the major areas in CMR where ML, and deep learning in particular, can assist clinicians and engineers in improving imaging efficiency, quality, image analysis and interpretation, as well as patient evaluation. We discuss recent developments in the field of ML relevant to CMR in the areas of image acquisition & reconstruction, image analysis, diagnostic evaluation and derivation of prognostic information. To date, the main impact of ML in CMR has been to significantly reduce the time required for image segmentation and analysis. Accurate and reproducible fully automated quantification of left and right ventricular mass and volume is now available in commercial products. Active research areas include reduction of image acquisition and reconstruction time, improving spatial and temporal resolution, and analysis of perfusion and myocardial mapping. Although large cohort studies are providing valuable data sets for ML training, care must be taken in extending applications to specific patient groups. Since ML algorithms can fail in unpredictable ways, it is important to mitigate this by open source publication of computational processes and datasets. Furthermore, controlled trials are needed to evaluate methods across multiple centers and patient groups.

**Supplementary information:**

**Supplementary information** accompanies this paper at 10.1186/s12968-019-0575-y.

## Introduction

**Fig. 1:**
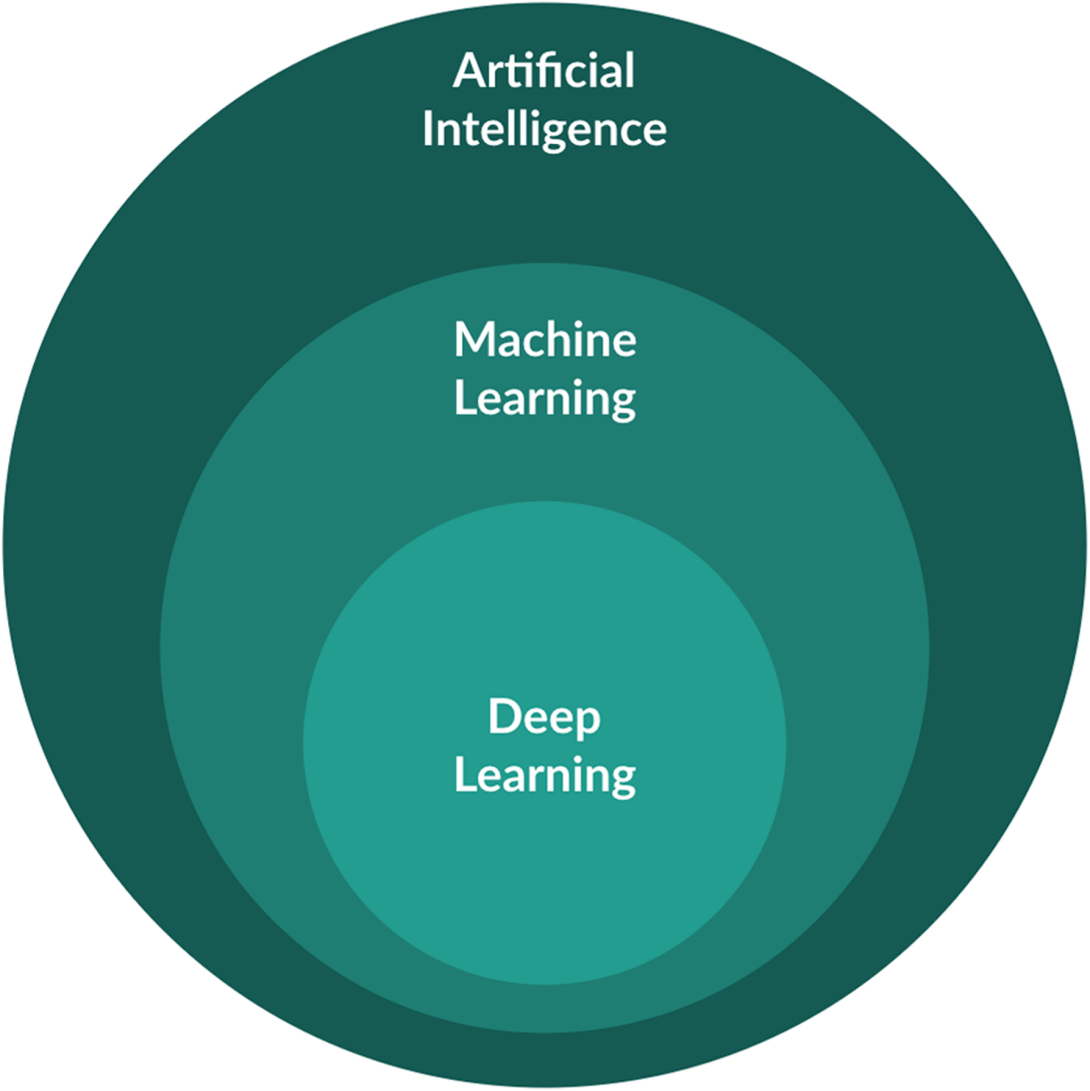
Artificial intelligence (AI) can be seen as any technique that enables computers to perform tasks characteristic of human intelligence. Machine learning (ML) is generally seen as the subdiscipline of AI which uses a statistical model together with training data to learn how to make predictions. Deep learning (DL) is a specific form of ML that uses artificial neural networks with hidden layers to make predictions directly from datasets

Machine learning (ML) and artificial intelligence (AI) are rapidly gaining importance in medicine [1, 2], including in the field of medical imaging, and are likely to fundamentally transform clinical practice in the coming years [3, 4]. AI refers to the wider application of machines that perform tasks that are characteristic of human intelligence, e.g. infer conclusions from deduction or induction, while ML is a more restricted form of computational processing which uses a mathematical model together with training data to learn how to make predictions. Rather than explicitly computing results from a set of predefined rules, ML learns parameters from examples and therefore has the potential to perform better at a task such as detecting and differentiating patterns in data by being exposed to a more examples. The most advanced ML techniques, also called deep learning (DL), are especially well-suited for this purpose (Fig. 1). Cardiovascular magnetic resonance (CMR) is a field that lends itself to ML because it relies on complex acquisition strategies, including multidimensional contrast mechanisms, as well as the need for accurate and reliable segmentation and quantification of biomarkers based on acquired data, to help guide diagnosis and therapy management.

It is important for clinicians and researchers working in CMR to understand the impact of ML on the field. Thus, the purpose of this review is threefold: firstly, we will provide a non-technical overview of the basics of ML relevant to CMR. Secondly, we survey the various ways ML has been applied to the field of CMR. Finally, we provide an outlook on future directions and recommendations for reporting results. Please also refer the glossary of terms for definitions of commonly used terms in machine learning.

### Machine learning basics

Consider the problem of reporting left ventricular (LV) ejection fraction (LVEF) from a CMR study. A traditional image processing method would typically define a sequence of steps a priori, e.g. selection of end diastolic (ED) and end systolic (ES) frames, contouring of cavity and myocardium using signal processing algorithms with a sequence of processing steps, calculation of cavity area per slice, summation into volumes, and the calculation of LVEF. In comparison, a ML method would learn from a set of examples, e.g. hundreds of CMR studies with ground truth segmentations, to optimize a mathematical model which is then used to predict segmentations. In this case the algorithm learns which parts of the data are important for the task, and how to put the information together to produce the result.

#### Standard machine learning models

In standard ML model, important characteristics or *features* for performing a certain task are extracted from images by using a designed feature set. In the example above, features for myocardial contours may include image contrast, noise characteristics, texture and motion. Once the design process is complete, ML methods need to be trained using example data. In this *training phase*, parameters of the feature set model are learned. A model is any function of the features used for prediction and the parameters of the model dictate the actual predictions made. Once trained, the model can be used to make a prediction for data not seen previously in the training phase. ML models can perform either *classification* where discrete labels such as the presence or absence of disease are determined, or *regression*, where continuous variables such as T1 are estimated. Because the models learn from examples, it is important that a sufficiently large dataset with representative variability is available for training. For evaluation of the model’s performance it is of utmost importance to keep training data that is used during model development and fine-tuning separated from the test data that is used to evaluate the model’s performance. Another dataset (usually called the *validation* dataset), is used during the training phase to help determine the optimal design of the ML model. This dataset is used to optimize model parameters, and to ensure that the model does not overfit.

#### Deep learning

One of the key steps in creating ML systems is designing the optimal discriminative features for a given task. This has proven highly challenging [5, 6]. A subfield of machine learning that can address this challenge is DL. Unlike standard ML methods, DL methods are able to learn directly from the data, circumventing the need for hand-crafting of discriminative features. In the example of finding the contours of myocardium, DL methods learn the image features most useful for predicting the location of the contours.

Recent successes with DL have been fueled by four synergistic advances: 1) the availability of large quantities of high-quality digital image data for training; 2) the ability of algorithms to learn relevant information directly from images without the need for handcrafted features; 3) low-cost powerful graphics processing unit (GPU) hardware, and 4) open source development libraries and working example networks made freely available by companies and researchers. These advances have led to the development of neural networks with many layers, which is what ‘deep’ refers to in DL [7]. A special type of DL network, the *convolutional neural network* (CNN) is often used for image analysis tasks.

**Fig. 2:**
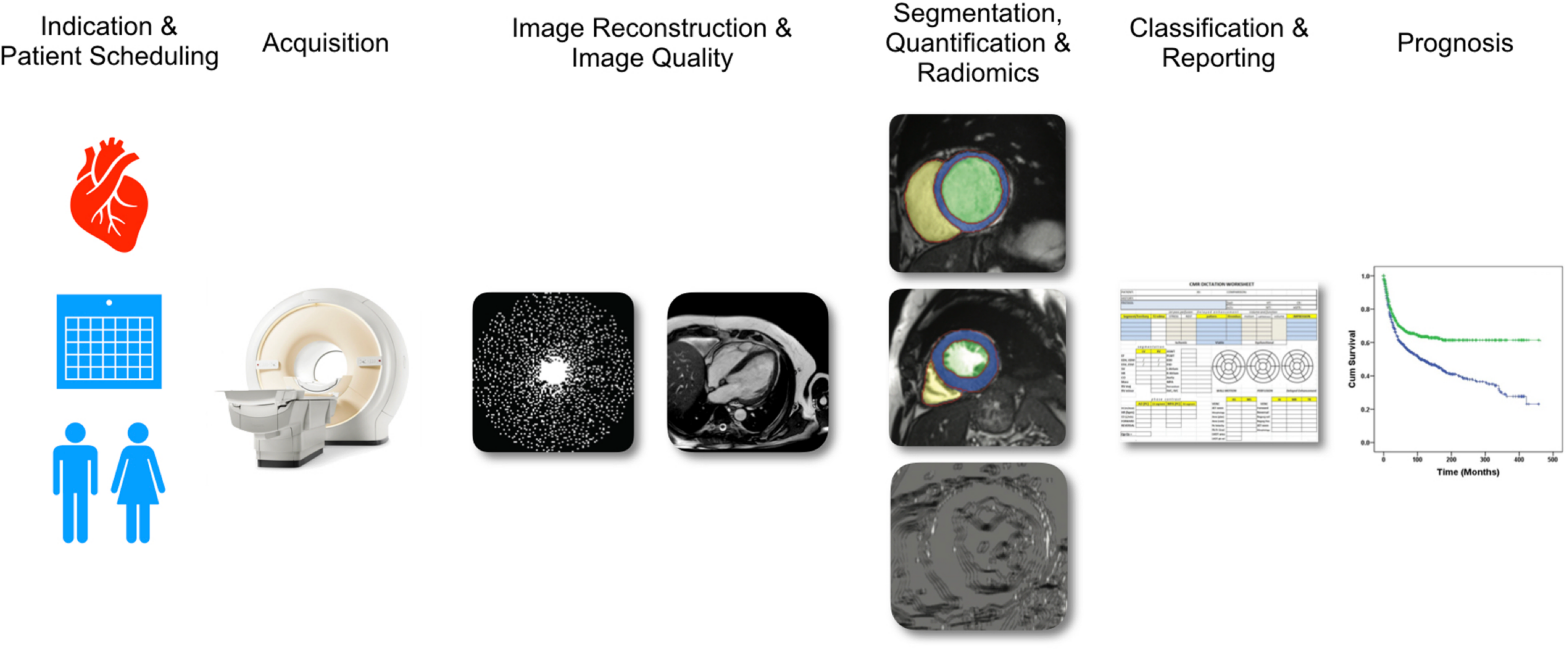
Machine learning will impact all aspects of cardiovascular magnetic resonance imaging from patient scheduling to image analysis and prognosis

A typical CNN network is composed of multiple layers, each with a well-defined architecture (Fig. 2). *Convolution layers* refer to those which employ a set of filters that are applied to the image to produce spatially dependent features for the next layer. The intent is to learn the optimal values of the filters (also called *weights*) so that features of maximum relevance to the task are generated in the subsequent layers. *Pooling layers* (e.G. *max* pooling or average pooling) downsample the spatial information so that features become more canonical for the task. For classification and some regression networks, a *fully connected layer* is used in which each node is connected to all other nodes in the layer. Segmentation networks often use upsampling operations to return the image dimensions back to the input image size. *Skip layers* are often employed, which enable the propagation of fine details from one layer to another, with the intent of recovering fine imaging features and improving gradient propagation during training. Finally, a *softmax layer* performs a non-linear function which rescales the components to give a non-negative probability to each pixel class. This ensures that outputs sum up to 1 in the output layer. Often deep CNN implementations contain many millions of weights. Although the features resulting from the convolutions in the intermediate layers contain information pertinent to the task, it is often difficult to interpret how the network makes its predictions, or why it failed. However, DL is currently the most popular ML architecture for medical image analysis. A recent survey [8] shows more than 300 DL papers have been contributed to the medical image analysis field, including CMR, in the 6 years to 2017, with the numbers growing exponentially.

#### Supervised and unsupervised learning

Based on the availability of reference labels in the training data, ML algorithms are commonly divided into *supervised* and *unsupervised* learning. In supervised learning, training data are accompanied with ground truth labels, e.g. cases with pathological status, images with expert-drawn contours, or cases with cardiac volume and function measurements. Supervised learning is the most commonly used approach in ML because learning from expert-annotated labels is the most intuitive way to mimic human performance. This is in contrast to unsupervised learning where training data are given without labels. Unsupervised learning is more challenging for building a prediction model, because it is closer to natural learning by discovering structures through observation [7]. Currently, a typical use of unsupervised learning is to explore hidden structure inside the training data. An example relevant to CMR is the work by Oksuz et al. who used unsupervised dictionary-based learning to segment myocardium from cine blood oxygen level dependent (BOLD) CMR [9].

ML model parameters can be estimated by assuming a simple functional relationship between the data and the labels, for instance between CMR images and a certain diagnosis. A classic example is linear discriminant analysis, which learns to fit a hyperplane to the training data by optimizing linear coefficients, e.g. to separate patients with reduced LVEF from subjects with normal LVEF. However, typical problems are complex and multi-dimensional with a large amount of data, and simple mathematical relationships cannot be assumed. Alternatively, ML model parameters can be optimized by an iterative process designed to refine the model behavior under some regularization constraints. Regularization is a mathematical tool to take into account prior information when solving an optimization task. Examples are support vector machines, e.g. applied to characterize vessel disease from intravascular images [10], random forests e.g. applied for T2 map quantification [11]) and DL CNNs, which is the focus of this review.

### Current applications of machine learning in CMR

#### Image acquisition and reconstruction

Efficient, high-quality CMR demands careful attention to proper patient positioning as well as planning of imaging planes and volumes [12]. In current clinical practice, CMR examinations are therefore performed by highly experienced operators. Several of these acquisition-related aspects of the CMR examination, which are currently performed manually on most commercial CMR systems, can be either automated or substantially shortened using ML. Multiple CMR hardware vendors are working on workflow optimizations such fully automated localization of the heart and planning of image acquisition planes aligned with the principal cardiac axes [13, 14]. Other investigators have applied ML to automate optimal frequency adjustment for CMR at 3 T [15], and to create a scan control framework that detects image artifacts during the scan and self-corrects imaging parameters or triggers a rescan if the prediction indicates the current slice has artifacts [16].

**Fig. 3:**
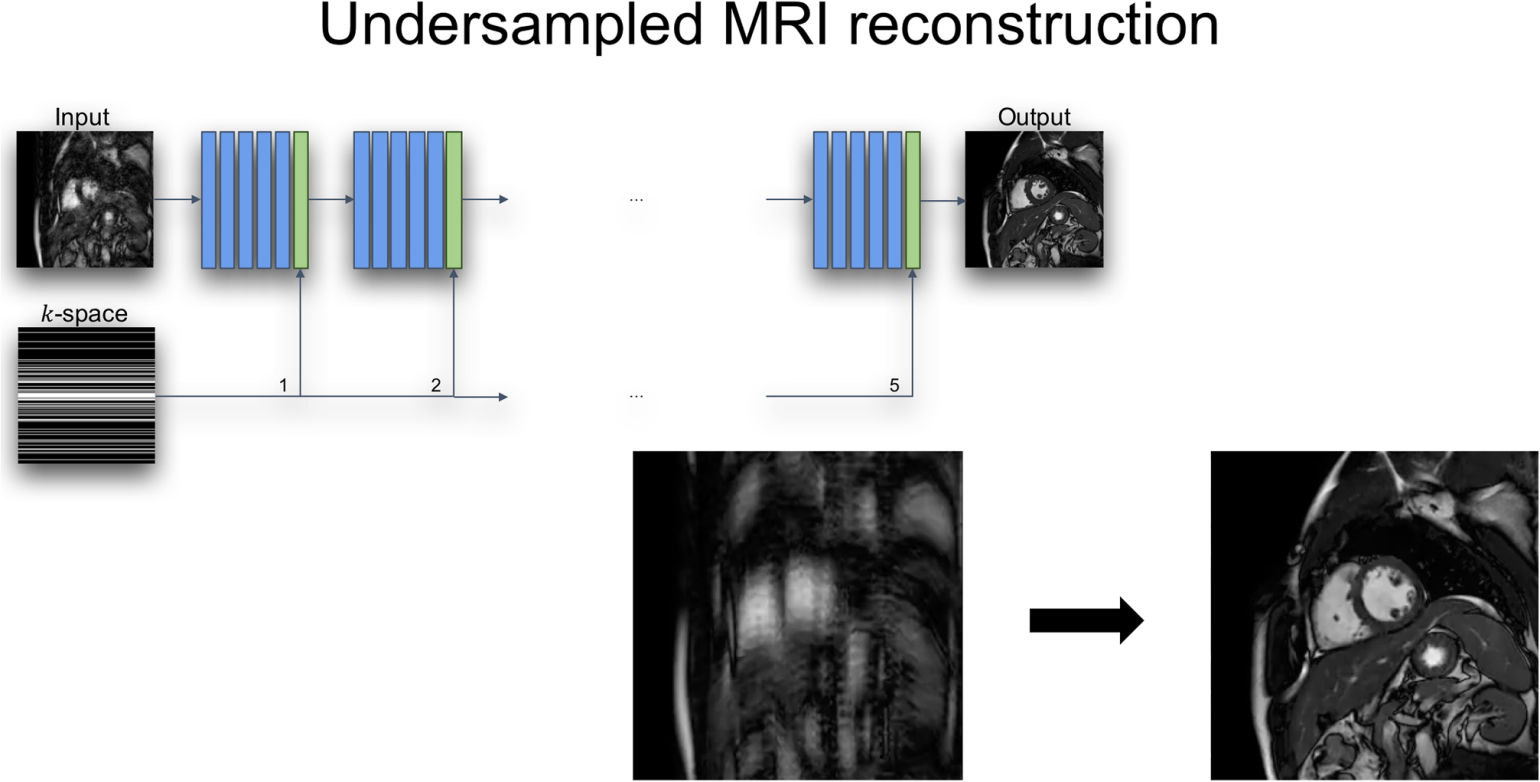
Deep learning network for reconstruction of undersampled CMR images

While CMR imaging offers a range of advantages for assessment of cardiac structure and function, acquisition of CMR images is slow as it is complicated by cardiac and respiratory motion. This imposes significant demands on patients (e.g. in terms of length of scan time and length of breath-holds) as well as making CMR expensive and less accessible. Over the last decade approaches such as parallel imaging and compressed sensing (CS) as well as real-time imaging have been increasingly employed to accelerate the acquisition of CMR images [17–26]. Techniques such as CS are particularly attractive for accelerating CMR as they undersample k-space, thereby leading to faster image acquisition. CS techniques such as [27–29] can be regarded as ML methods that exploit spatiotemporal redundancies in CMR data to learn how to recover an uncorrupted image from undersampled k-space measurements. For this, CS techniques exploit the sparsity (or *compressibility*) of CMR images. More recently DL techniques have emerged that use convolutional neural networks in order to replace the generic sparsity model used in CS techniques with a model that is learnt from training data [30, 31]. An advantage of these DL approaches is that they not only offer superior performance in terms of reconstruction quality but that they also offer high efficiency, e.g. very fast reconstruction speeds, making clinical deployment feasible [31] (Fig. 3).

**Fig. 4:**
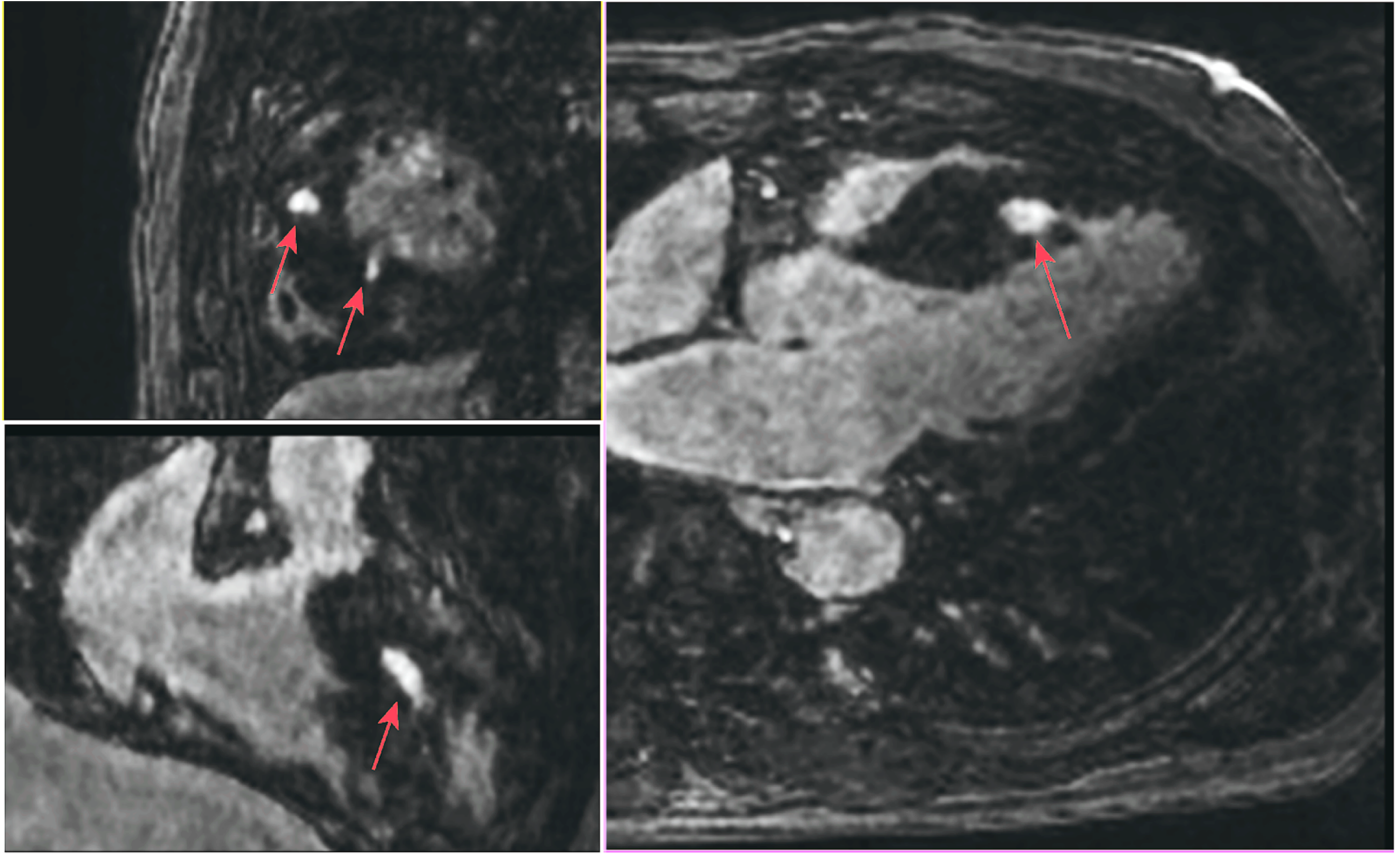
Late gadolinium enhancement (LGE; red arrows) images with isotropic spatial resolution of 1.4 mm^3^ reconstructed using deep learning from a prospectively five-fold randomly undersampled 3D LGE dataset in a patient with hypertrophic cardiomyopathy

More recently, DL approaches that exploit spatiotemporal redundancy via recurrent CNNs have been proposed which are more compact than cascades of CNNs [32]. A remaining challenge is the integration of DL approaches with existing approaches for the acceleration of CMR, such as parallel or real-time imaging. Accelerated imaging is necessary in high-dimensional (e.g. 3D or 4D) imaging for late gadolinium enhancement (LGE), flow or perfusion imaging. DL techniques have the potential to be applied to reduce the reconstruction time of highly accelerated 3D or 4D dataset. Figure 4 shows an example 3D LGE image reconstructed using DL with an acceleration factor of 5.

#### Image segmentation

Delineating the borders of the chambers and myocardium (a process known as segmentation) is mandatory in CMR post processing [33], but it is a time-consuming task. Experienced readers may produce high precision manual contours, but differences among expert readers are still known to occur [34]. A large body of research has been dedicated to developing automated CMR segmentation methods (see reviews in [35, 36]), but manual corrections are still needed in the areas where there are lots of trabeculae, the LV outflow tract, apical slices, as well the right ventricle. ML algorithms can be very helpful to further automate this task to increase the productivity of CMR segmentation while improving accuracy and reproducibility [37] over the techniques described in the two reviews mentioned earlier [35, 36]. In general, DL-based fully automated LV segmentations are highly accurate with 9 out of 10 recently developed methods [37] achieving Dice similarity coefficients of 0.95 or better.

DL frameworks developed for general image segmentation can be applied directly to segment the myocardium and cardiac chambers from CMR images, often by using pixel-based classification. Many reports have been based on the U-Net architecture [38]. For instance, a basic CNN layout with 9 convolutional layers and a single upsampling layer was used to segment short-axis CMR images [39]. A fully convolutional approach with a simpler upsampling path has been suggested by Bai et al. [40] and successfully applied for pixelwise segmentation of 4-chamber, 2 chamber and short axis CMR images in less than 1 min. Contextual 3D spatial information can also be integrated in the CNN architecture by providing features learnt from adjacent slices [41] or detecting a canonical view before segmentation [42]. Several studies have combined CNN with other ML algorithms, such as constraining the optimization process by constraining the network with information about the shape of the heart [43] or using the output of the DL model as the initial template for a deformable model segmentation [44, 45].

**Fig. 5:**
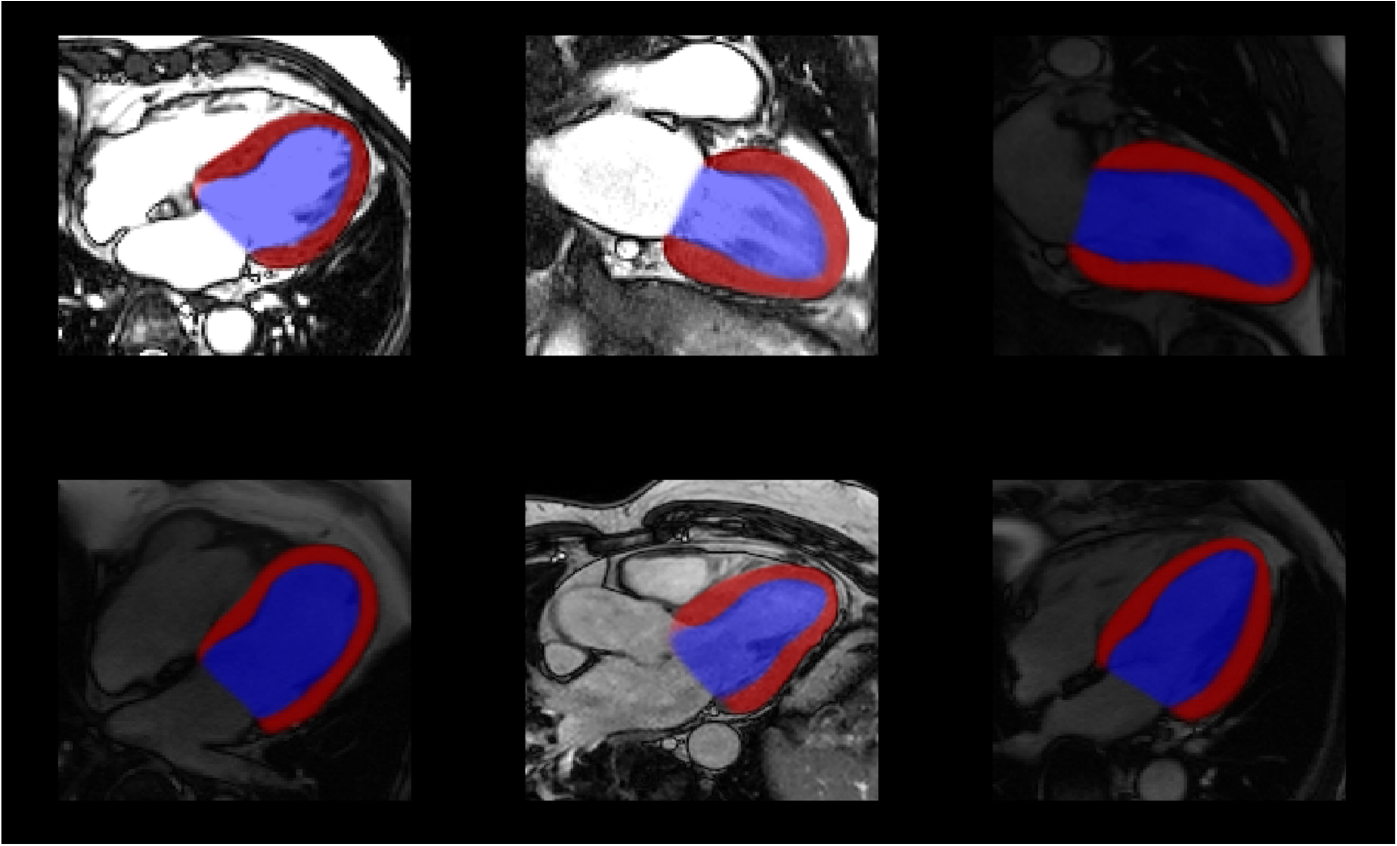
Some examples of deep learning based myocardial segmentation on long-axis CMR images, trained from almost 5000 cases. A U-Net network architecture was used in this case to classify myocardium (red) and cavity (blue)

**Fig. 6:**
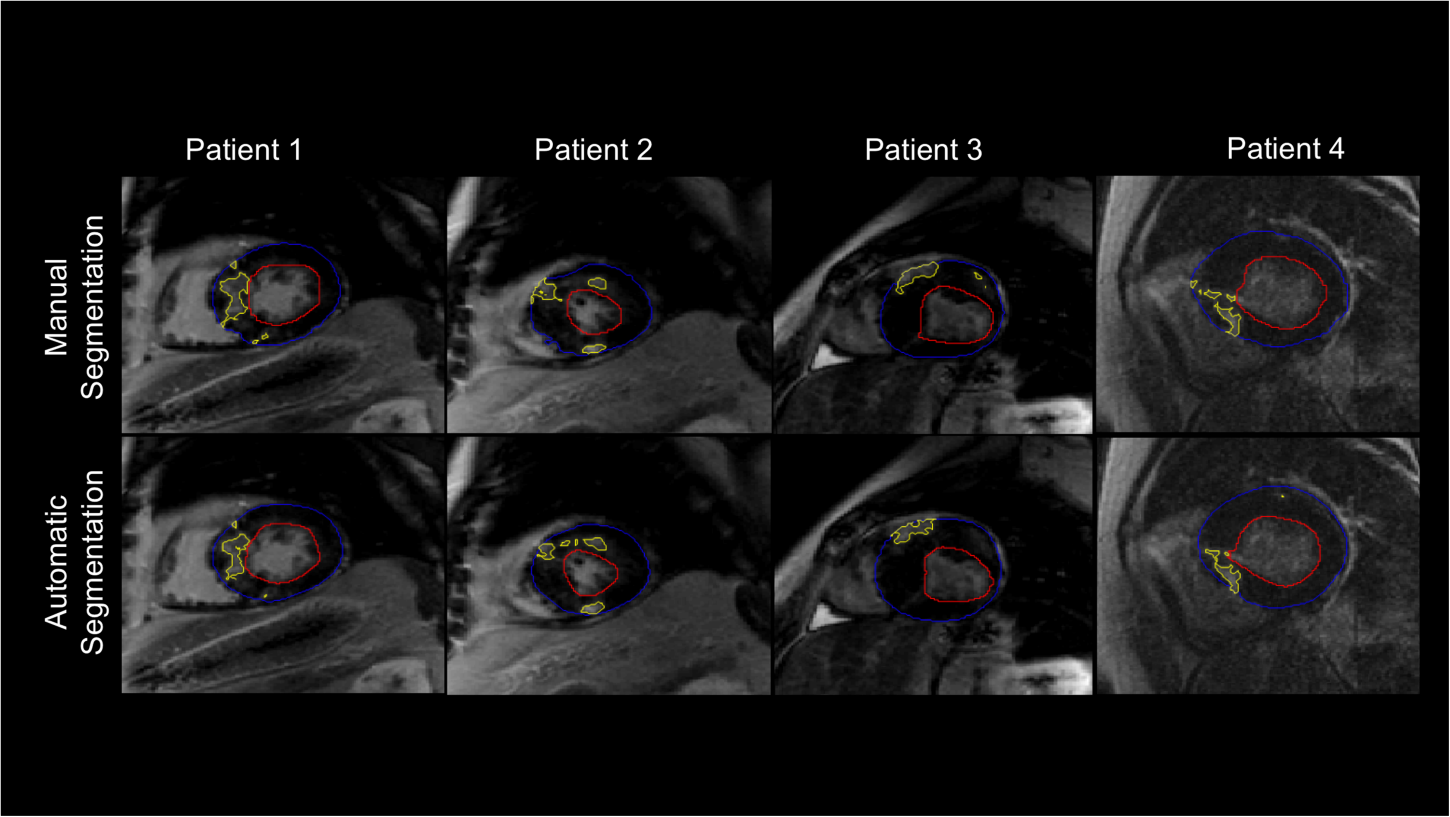
Scar and myocardium segmentation results for slices from four different patients. Contours resulting from manual (top row) and automatic (lower row) segmentations for the epicardium (blue), endocardium (red), and scar (yellow) boundaries are overlaid on late gadolinium enhancement (LGE) images

A different approach in DL segmentation is to perform regression rather than pixel classification. In [46], a network was trained to automatically identify myocardium and detect the center of the cavity. Then another network was trained to estimate radii from the cavity center, producing smooth epicardial and endocardial contours. A similar approach was also proposed by [47], where a boundary regression was performed on both left and right ventricles on short-axis images producing contours instead of pixel classification. Examples of image segmentation based on DL are shown in Figs. 5 and 6.

DL methods can also calculate functional parameters from imaging, e.g. fully automated determination of LVEF, which subsequently can be used as a basis to triage patients into different disease categories using handcrafted features [35, 48]. Puyol-Antón et al. [49] have taken this approach a step further and used a database of CMR and cardiac ultrasonography images as well as clinical information to design a ML-based diagnostic algorithm that can fully automatically identify patients with dilated cardiomyopathy using a support vector machine.

#### Myocardial tissue characterization

ML has been applied to a variety of myocardial tissue characterization tasks. For example, scar volume from LGE CMR is a quantitative imaging biomarker with inherent prognostic information, where application of ML allows to overcome the need for subjective, time-consuming and labor-intensive manual delineation currently used in routine clinical practice. Even when using the current thresholding techniques for LGE quantification, accuracy and reproducibility remain a major challenge due to variations among different CMR centers [50], variations in gadolinium kinetics, and the patchy, multifocal appearance of LGE, e.g. in patients with hypertrophic cardiomyopathy (HCM). To address these shortcomings, a novel, ML-based approach to LGE quantification has recently been suggested by Fahmy et al. [51].

**Fig. 7:**
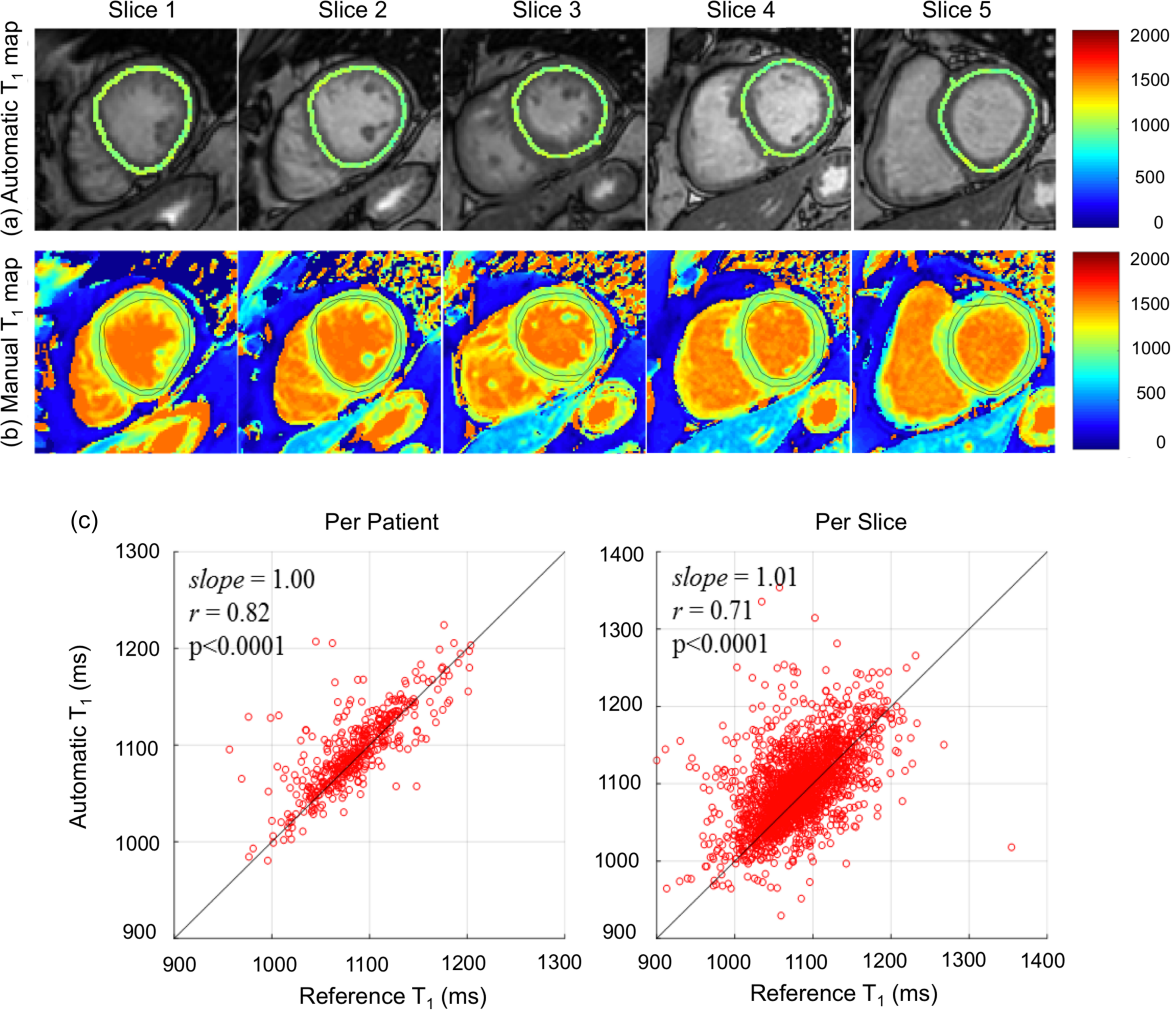
Myocardial T1 mapping at five short axial slices (apex to base from left to right respectively) of the left ventricle of one patient. **a** Automatically reconstructed map (after automatic removal of myocardial boundary pixels) overlaid on a T1 weighted image with shortest inversion time; (**a**) Manually reconstructed T1 map. The contours in (**b**) represent the myocardium region of interest manually selected by the reader. In Fig. **c** scatter plots are shown of the automatic versus manual myocardium T1 values averaged over the patient volume (left) and each imaging slice (right). Solid lines represent the unity slope line

Cardiac relaxometry has demonstrated capabilities for further quantitative tissue characterization. For example, T1 mapping has proven beneficial for the identification of diffuse myocardial fibrosis as well as myocardial edema and lipid deposition. While there have been numerous reports on data acquisition, little attention has been paid to data analysis and reporting. Recent work has shown that ML can also be applied to streamline data processing and analysis for myocardial tissue characterization [51–55] (Table 1 and Fig. 7).

**Table 1:** Machine learning and deep learning for LGE quantification and parametric mapping

Author	Myocardial disease	Image substrate	Application
Fahmy et al., 2018 (Ref. 51)	HCM	LGE	Delineate and quantify scar volume in patients with HCM
Hann et al., 2018 (Ref 52)		T1 mapping	Automated LV segmentation of T1 maps using a ShMOLLI sequence in order to speed up LGE quantification based on T1 mapping
Fahmy et al., 2019 (Ref 53)	Various diseases	T1 mapping	DL based image analysis and motion correction for myocardial T1 mapping to provide fast and automated T1 mapping analysis (DICE: 0.85)
Farrag et al., 2019 (Ref 55)	Myocardial infarction	T1 mapping and CINE	DL based automated LV segmentation of T1 maps using a ShMOLLI sequence (DICE: 0.84)
Martini et al., 2018 (Ref 54)	Various diseases	T1 mapping	Automated segmental analysis of T1 maps (DICE: 0.98, Jaccard: 0.97)

*ML* machine learning, *DL* deep learning, *HCM* hypertrophic cardiomyopathy, *LGE* late gadolinium enhancement, *shMOLLI* shortened modified Look-Locker inversion recovery

**Fig. 8:**
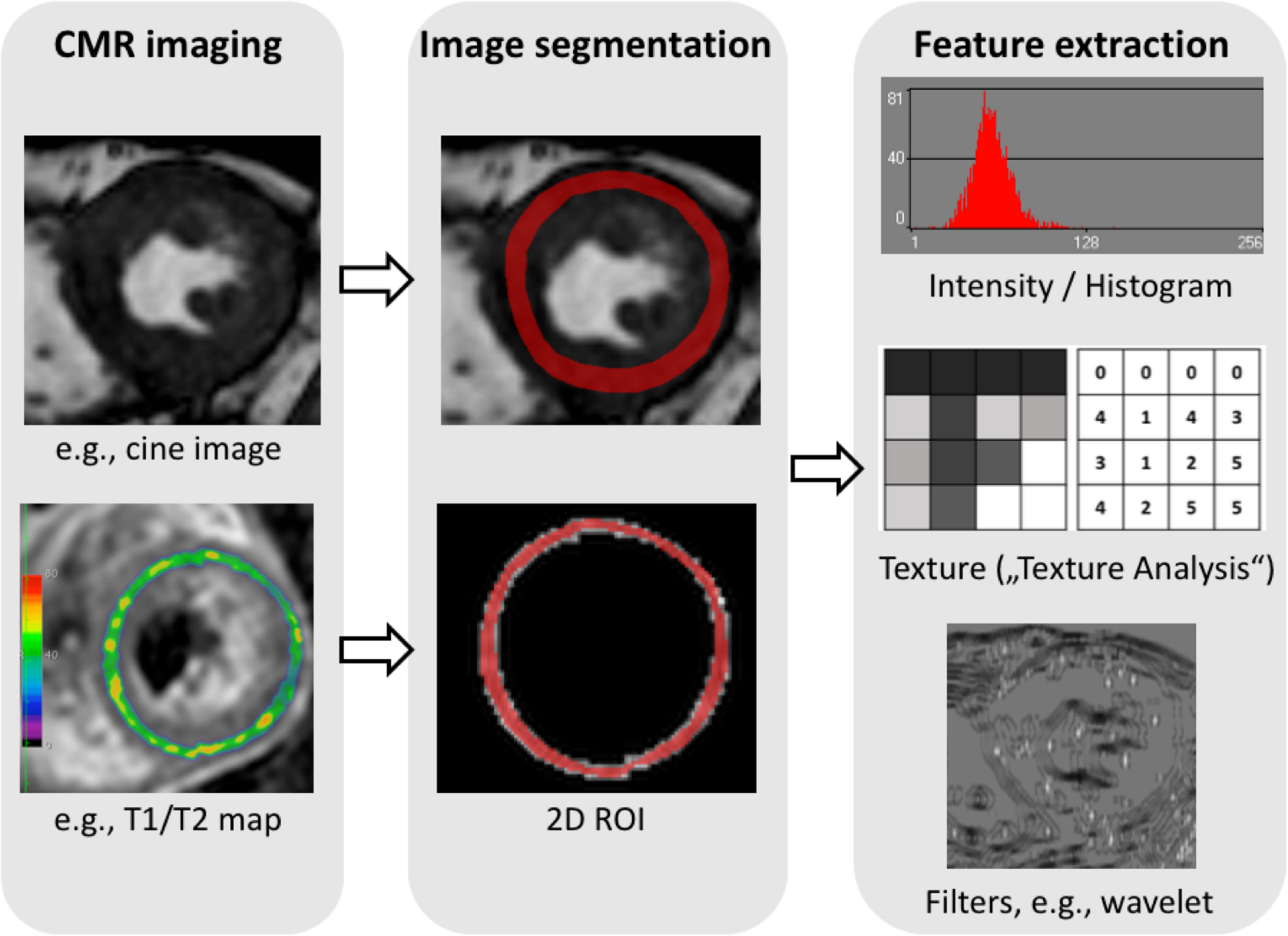
Radiomics in CMR. Radiomic feature extraction can be performed on all types of CMR images, e.g. cine images or T1 / T2 maps. The myocardium is segmented either manually or automatically using DL algorithms and feature extraction is performed. Whereas shape features are of high interest in oncologic imaging, radiomics in CMR mostly rely on intensity based / histogram, texture features and filter methods such as wavelet transform. After extracting a high number of quantitative features from CMR images, high-level statistical modelling involving ML and DL methods is applied in order to perform classification tasks or make predictions in a given dataset

The ability of ML techniques to cope with high-dimensional data has recently facilitated the exponential growth of a novel field called *radiomics*. The term radiomics reflects a process of converting digital medical images into mineable high-dimensional data [56] by extracting a high number of handcrafted quantitative imaging features based on a wide range of mathematical and statistical methods. Various features can be extracted from images, the most important being morphologic, intensity-based, fractal-based, and texture features (subsumed under the term “texture analysis” [TA]) [57]. Texture features model spatial distributions of pixel grey levels and allow for the segmentation, analysis and classification of medical images according to the underlying tissue textures [58], thus offering the potential to overcome limitations of a pure visual image interpretation [59] (Fig.8).

Although radiomics and TA have been applied most prominently in the fields of oncologic and neurologic imaging, the first applications have been described for CMR. Since myocardial tissue characterization remains an important but complex and challenging task for differentiating amongst various cardiac diseases, the application of radiomics to CMR imaging data appears to be appealing in order to deliver further insights into the complex tissue changes and pathology of cardiovascular diseases.

The first applications of radiomics and TA in CMR have been reported for segmentation of scarred tissue areas in myocardial infarction [60–62], allowing for enhanced visualization of scarred myocardium and extracting information about the characteristics of the underlying myocardial tissue (Table2). Since then, several studies have been published, showing the feasibility of TA to differentiate between acute and chronic infarction [63], based either on a combination of non-contrast cine and LGE imaging [64], or based on cine imaging alone [59, 65, 73].

**Table 2:** Radiomics and texture analysis in CMR

Author	Myocardial disease	Image substrate	Application
Beliveau, P. et al., 2015 (Ref 63)	Myocardial fibrosis (rat model)	LGE	Detection of age-related myocardial fibrosis (correlation to histopathology)
Engan, K. et al., 2010 (Ref 60)	Myocardial infarction	LGE	Discrimination of patients with low risk of arrhythmias from those with high risk of arrhythmias
Kotu, L.P. et al., 2013 (Ref 61)	Myocardial infarction	LGE	Automated segmentation of scarred tissue areas
Kotu, L.P. et al., 2013 (Ref 62)	Myocardial infarction	LGE	Enhanced visualization and segmentation of scarred myocardium
Larroza, A. et al., 2017 (Ref 64)	Myocardial infarction	LGE, Cine	Differentiation between acute and chronic MI (AUC 0.86 for LGE, 0.82 for Cine)
Baeßler, B. et al., 2018 (Ref 53)	Myocardial infarction	Cine	Differentiation between normal myocardium and small (AUC 0.92) as well as large scar (AUC 0.93)
Larroza, A. et al., 2018 (Ref 65)	Myocardial infarction	Cine	Differentiation between nonviable, viable, and remote myocardial segments; extraction of TA features over the entire cardiac cycle; AUC 0.85
Schofield, R. et al., 2016 (Ref 66)	Hypertrophic heart (hypertrophic cardiomyopathy, amyloid, aortic stenosis)	Cine	Differentiation amongst several causes of myocardial hypertrophy (HCM, amyloid and aortic stenosis) and healthy controls
Thornhill, R.E. et al., 2014 (Ref 67)	Hypertrophic cardiomyopathy	LGE	Differentiation between segments with and without hypertrophy and fibrosis
Baeßler, B. et al., 2018 (Ref 68)	Hypertrophic cardiomyopathy	Native T1-weighted	Differentiation between HCM patients and controls (AUC 0.95)
Cheng, S. et al., 2018 (Ref 69)	Hypertrophic cardiomyopathy	LGE	Association of adverse events in HCM patients with systolic dysfunction with increased LGE heterogeneity
Neisius, U. et al., 2018 (Ref 70)	Hypertensive heart disease, hypertrophic cardiomyopathy	Native T1 map	Discrimination between hypertensive heart disease and HCM patients with incremental value over global native T1 mapping
Baeßler, B. et al., 2018 (Ref 71)	Acute myocarditis	Native T1 mapping, T2 mapping	Diagnosis of biopsy-proven acute infarctlike myocarditis (AUC 0.88)
Baeßler, B. et al., 2017 (Ref 72)	Dilated cardiomyopathy-like myocarditis	Native T1 mapping, T2 mapping	Diagnosis of biopsy-proven acute myocarditis presenting with symptoms of heart failure

Besides infarction, other applications of TA and radiomics have recently been reported for CMR. Several smaller studies demonstrated the use of texture features for differentiating amongst several causes of myocardial hypertrophy (i.e. HCM, amyloid and aortic stenosis) and healthy controls [74], or to detect fibrosis in HCM patients [66, 67]. Cheng et al. [68] evaluated the prognostic value of texture features based on LGE imaging in HCM patients with systolic dysfunction, demonstrating that increased LGE heterogeneity was associated with adverse events in HCM patients with systolic dysfunction. Recently, TA has been applied to native T1mapping for discriminating between hypertensive heart disease and HCM patients, providing incremental value over global native T1 mapping [69].

Myocardial inflammation is another interesting topic, where radiomics and TA are extremely appealing in order to overcome the current limitations of qualitative as well as novel quantitative CMR sequences [11, 75]. Recent work has shown that averaging T1 and T2 values derived from T1 and T2 mapping over the entire myocardium has low sensitivity and specificity for detecting myocardial inflammation [11, 72, 75], and that analysis of inflammation-induced tissue inhomogeneity on T1 and/or T2 maps might enable more accurate quantification of myocardial inflammation [11, 71, 76]. Very recently, a first application of radiomics on T1 and T2 mapping in a cohort of patients with biopsy-proven acute infarct-like myocarditis has demonstrated an excellent diagnostic accuracy of TA [70] and the concept has also been shown to be applicable to the much more challenging diagnosis of chronic myocardial inflammation or myocarditis presenting with heart-failure symptoms [77].

#### Prognosis

Information in CMR images obtained for diagnostic purposes can also be used for prognosis. In a meta-analysis of 56 studies containing data of 25,497 patients with suspected or known coronary artery disease (CAD) or recent myocardial infarction, El Aidi et al. [78] found that LVEF was an independent predictor of future cardiovascular events; predictors for patients with suspected or known coronary artery disease (CAD) were wall motion abnormalities, inducible perfusion defects, LVEF, and presence of infarction. Although meta-analyses such as these can help identify imaging features important for prognosis, selection of potentially relevant features is a manual process based on presumed pathophysiological importance and the ability to easily and reproducibly quantify parameters of interest. Another important limitation is that ‘traditional’ meta-analysis often fails to capture the heterogeneity between studies and patients in sufficient detail to establish the association between CMR findings and outcome. Furthermore, in almost all studies just a small fraction of all available information about individual patients is taken into account. Machine learning, on the other hand, is ideally suited in finding intrinsic structure within patient phenotypic data containing a high number (i.e. hundreds or even thousands) of variables, which can then be evaluated both retrospectively and prospectively for predicting outcomes. Thus, ML is much better suited for dealing with the high-dimensionality of ‘real-world’ datasets and can be used for *unbiased* identification of prognostically important variables. ML is also ideally suited to incorporate information from electronic health records (EHR), laboratory data and genetic analyses.

Machine learning for prediction of adverse cardiovascular events has the potential to augment of traditional risk scores, developed from the Framingham Heart Study and other large cohort studies, with novel biomarkers derived from imaging methods. In the Multi-Ethnic Study of Atherosclerosis (MESA), 6814 initially asymptomatic participants were followed for over 12 years. Over 700 variables were collected. Ambale-Venkatesh et al. [79] used a random survival forests technique to identify the top-20 predictors of each outcome measure. In addition to carotid ultrasound as a predictor for stroke, and coronary calcium score as a predictor of atherosclerotic cardiovascular disease, CMR derived LV structure and function were among the top predictors for incident heart failure [79]. The random survival forest risk prediction performed better than established risk scores with increased prediction accuracy. Another application is in dimension reduction methods, which show promise in the detection of multidimensional shape features to characterize ventricular remodeling. Mass, volume and univariate measures such as sphericity have shown prognostic value in MESA [80]. Zhang et al. [81] applied information maximizing component analysis to determine shape features which best characterize differences between patients with myocardial infarction and asymptomatic volunteers.

ML has also been used to predict outcome in patients with cardiovascular disease. In a small proof-of-concept study, Kotu et al. [82] used supervised ML to predict the occurrence of cardiac arrhythmia in patients who survived infarction. The investigators found that CMR-derived scar texture features based on scar gradient and local binary patterns along with information about size and location of the scar demonstrated discriminative power for risk stratification comparable to currently used criteria such as LVEF and scar size. Finally, Bello et al. [83] used CMR-derived features together with clinical information to train a DL classifier that can predict outcome in patients with pulmonary hypertension. A dense motion model was used to identify patterns of right ventricular (RV) motion associated with adverse outcomes, with superior results to prognostication based on RV ejection fraction or strain.

ML methods have also been used to quantify relationships between cardiac morphology and genetic variations. For example, Schafer et al. found associations between titin-truncating variants and concentric remodeling in healthy individuals [84]. Mass univariate models were used to find associations with single nucleotide polymorphisms (SNPs) in genome-wide association studies (GWAS) studies [85]. Work by Peressutti et al. [86] has shown that ML could be used to identify patients with a favorable response to cardiac resynchronization therapy (CRT) by supervised learning of relationships between cardiac motion abnormalities, EKG data, clinical information and the success of CRT as assessed at 6-months follow-up. The same group of investigators has also demonstrated that more detailed analysis of myocardial strain at multiple different anatomical scales can be used to refine prediction of CRT response [87].

## Barriers to implementation

Although ML and DL are powerful new methods which can help optimize the entire CMR imaging value chain, there are also several limitations that need to be mentioned. The most important present limitations include difficulty with rare entities and rare presentations of common entities such as congenital heart disease. Other difficulties include reliance on small, fixed inputs with long-range or heterogenous dependencies such as medical charts, prior exams, and dealing with multiple CMR imaging sequences and acquisition planes. Another limitation is the ‘black-box’ nature of DL algorithms since it is often unclear what information is used to come to a certain classification or result. Techniques to visualize salient features can potentially help address this limitation [88]. Furthermore, the present lack of model robustness and lack of portability with respect to different CMR scanners, sequences, imaging parameters and institutions need to be addressed. Another barrier in this regard is the lack of large, publicly available CMR datasets that can be used to objectively compare different (commercially available) algorithms with regard to their performance. Finally, many current ML and DL techniques are susceptible to adversarial attacks that may lead to erroneous results [89].

## Conclusions and future outlook

ML, and DL in particular, is beginning to be applied to different types of cardiac imaging [90]. Besides image interpretation, there are many tasks in the imaging process that can potentially benefit from application of ML. In the short term, ML techniques are highly likely to be incorporated in the image acquisition and reconstruction domains, in the postprocessing workflow and analysis of advanced image features beyond visually identifiable features as well as multi-dimensional contrasts and their interpretation. One promising method is to use DL methods to simulate images, both to augment the size and the variability in the training datasets for segmentation and classification networks and to characterize bias between different imaging modalities. A CMR scar simulation method has recently shown to improve identification of scar in LGE images [91]. Another promising technique is reinforcement learning, in which an agent is trained by trial and error using feedback from previous actions and experiences [92]

Despite the significant advances as described above there are currently no published clinical trials in which ML has been compared with human evaluation of CMR datasets. Prospective controlled clinical trials are required to establish the effectiveness of algorithms in clinical practice. The recently commenced CarDiac MagnEtic Resonance for Primary Prevention Implantable CardioVerter DebrillAtor ThErapy (DERIVATE) international observational registry is a good example of such a study [93]. Furthermore, validation must be performed not only using data from the same cohort as was employed in the training, but also from other cohorts. In particular, algorithms must be validated with data from different centers and different acquisition devices. An efficient way of subsequently comparing the performance of different algorithms is through so-called challenges – competitions where research teams evaluate their algorithms on a common dataset labeled with ground truth information, e.g. Kaggle platform [94] and grand challenge platform [95]. Ground truth also must be meticulously reviewed, in particular clinical reports since clinicians may disagree in reporting style (and findings) from center to center. Reported metrics are application dependent but need to include not only sensitivity and specificity but also positive predictive value and model metrics such as the area under the receiver operating characteristic curve. For the field to advance, algorithms should be published using open source repositories to enable replication, benchmarking, and improvement by other groups.

## Supplementary information

Additional file 1: Glossary of commonly used terms in Machine Learning. (DOCX 23 kb)

## Data Availability

Please contact author for data requests.

## Abbreviations

AI Artificial intelligence BOLD: Blood oxygen level dependent
CAD Coronary artery disease CMR: Cardiovascular magnetic resonance
CNN: Convolutional neural network
CRT: Cardiac resynchronization therapy
CS: Compressed sensing
DL: Deep learning
ED: End-diastolic
EHR: Electronic health records
ES: End-systolic
GLNU: Gray-level non-uniformity
GPU: Graphics processing unit
GWAS: Genome-wide association study
HCM: Hypertrophic cardiomyopathy
LGE: Late gadolinium enhancement
LV Left ventricular LVEF: Left ventricular ejection fraction
MESA: Multi-ethnic Study of Atherosclerosis
MI: Myocardial infarction
ML: Machine learning
RLNU: Run-length non-uniformity
RV Right ventricular ShMOLLI: Shortened modified Look Locker inversion recovery
SNP: Single nucleotide polymorphism
TA: Texture analysis
