# Supplementary material for: Machine learning in cardiovascular magnetic resonance: basic concepts and applications
Source: J Cardiovasc Magn Reson. 2019 Oct 7;21:61. doi: 10.1186/s12968-019-0575-y (PMC6778980; doi:10.1186/s12968-019-0575-y)
Supplement: Supplementary file 1 — Glossary of commonly used terms in Machine Learning. (DOCX 23 kb) [file 12968_2019_575_MOESM1_ESM.docx]

**Supplemental Material** – Glossary of commonly used terms in Machine Learning

| Artificial Intelligence (AI) | Machines that perform tasks that are characteristic of human intelligence. Computer science experts agree that the field is far away from universally applicable AI |
| --- | --- |
| Machine Learning (ML) | A form of computational processing which uses a statistical model together with training data to learn how to make predictions |
| Deep Learning | A specific form of ML that uses artificial neural networks with many hidden layers to learn features needed for prediction directly from a dataset |
| Supervised Learning | The process of deriving a ML model from examples annotated with ‘ground truth’ labels such as e.g. manually-drawn contours, a specific diagnosis or ejection fraction |
| Weakly Supervised Learning | The process of deriving a ML model from a partially or coarsely labeled dataset. This method is interesting because obtaining a ‘ground truth’ is can be very labor-intensive or costly |
| Unsupervised Learning | The process of deriving a ML model from unlabeled data, i.e. without a ground truth available. Unsupervised learning is primarily used to automatically identify structure in data |
| Transfer Learning | Reuse of an existing ML model to a another prediction task than the one it was designed for |
| Reinforcement learning | The process of deriving a ML model without training data, but the model is trained using an action, e.g. rotation of a 2D plane to show short-axis view from 3D volume image. The model is learnt by reward and punishment mechanism evaluated from the action result |
| Model | A mathematical description of how input data is converted into a prediction. Also called algorithm |
| Training | The process of using a dataset to search for a mathematical model that can perform a certain prediction task |
| Validation | The process of optimizing the ML model using data not previously seen in the training phase. Validation is used to ensure the model is not overfitted |
| Testing | The process of applying a ML model to unseen data to test the accuracy of performing a certain prediction task. Ideally, this dataset should be completely separate from the training and validation datasets |
| Overfitting | Creation of a ML model that is poorly generalizable to datasets other than the one it was trained on |
| Loss function | A mathematical function to quantify how far off the results predicted by the ML algorithm are from the expected result (i.e. it indicates the magnitude of error the ML model made on its prediction) |
| Regularization | A set of methods to discourage learning a too complex or flexible model, in order to avoid the risk of overfitting |
| Hyperplane | A subspace whose dimension is one less than that of its ambient space. Hyperplanes are used in support vector machines and  can be thought of as a mathematical function that describes the best possible ‘boundary’ between different categories of data |
| Support Vector Machines | A type of supervised ML model which constructs a hyperplane or set of hyperplanes in a high-dimensional space, which can be used for classification, regression, or other tasks like outliers detection |
| Random Forest | A commonly used supervised ML model that uses decision trees for classification or regression. The model is created from a training dataset by recursively splitting it smaller datasets that ‘best’ separate the data into the desired categories. Predictions are generated by averaging the results of a ‘going down’ a large number of trees |
| Convolutional Neural Network (CNN) | A specific form of deep learning loosely modeled on the neuronal structure of the biological brain. A CNN consists of multiple interconnected units or ‘neurons’ and is arranged in multiple layers. Every neuron within a layer receives its own copy of all the outputs of the previous layer as its input. The individual neurons can then signal other neurons upon receiving a specific input. Whether or not this happens depends on the activation function that is used. In the medical domain CNNs are powerful tools for classification tasks based on images. |
| Activation function | The mathematical function that describes the output of an individual node or ‘neuron’ of an artificial neural network given a specific input or set of inputs. An alternative name is the transfer function |
| Rectified linear unit (ReLU) | The most commonly used activation function in deep learning models. It returns a zero output if the input is negative, and the actual value of the input if the input is positive |
| Sigmoid activation function | An activation function that remains at zero until input is received, at which point the output increases quickly at first, but gradually levels off (i.e. approaches an asymptote) |
| Downsampling | Refers to the process of ‘filtering’ out the most important nodes in a layer of the CNN by applying a filter that keeps the average or maximum of a set of set outputs. Downsampling is an important step to reduce the amount of data to work with to reduce the computational burden as well as to avoid overfitting. Also called pooling. |
| Upsampling | The process of creating an output with higher resolution compared to the input, e.g. an image with a larger matrix of pixels. |
| Kernel | Refers to the sets of learnable parameters applied in convolution operations. |
| Softmax | Refers to a non-linear function in which negative outputs are rectified. This ensures that outputs sum up to 1 in the output layer. |
| Graphics processing unit (GPU) | A type of computer chip specifically designed for performing the same operation over and over very quickly in very large datasets. This is in contrast to conventional central processing units (CPU), which tend to perform these operations much slower. |
| Radiomics | Refers to a set of techniques whereby digital medical images are seen as mineable high-dimensional data. From this data a high number of handcrafted quantitative imaging features based on a wide range of mathematical and statistical methods can be extracted. The extracted data can be used as input for conventional machine learning classifiers, such as random forests and support vector machines. |
